# Frequency-induced negative magnetic susceptibility in epoxy/magnetite nanocomposites

**DOI:** 10.1038/s41598-021-82590-w

**Published:** 2021-02-08

**Authors:** Che-Hao Chang, Shih-Chieh Su, Tsun-Hsu Chang, Ching-Ray Chang

**Affiliations:** 1 Interdisciplinary Program of Sciences, National Tsing Hua University, Hsinchu, Taiwan; 2 Department of Physics, National Tsing Hua University, Hsinchu, Taiwan; 3 Department of Physics, National Taiwan University, Taipei, Taiwan

**Keywords:** Electronic properties and materials, Magnetic properties and materials

## Abstract

The epoxy/magnetite nanocomposites express superparamagnetism under a static or low-frequency electromagnetic field. At the microwave frequency, said the X-band, the nanocomposites reveal an unexpected diamagnetism. To explain the intriguing phenomenon, we revisit the Debye relaxation law with the memory effect. The magnetization vector of the magnetite is unable to synchronize with the rapidly changing magnetic field, and it contributes to diamagnetism, a negative magnetic susceptibility for nanoparticles. The model just developed and the fitting result can not only be used to explain the experimental data in the X-band but also can be used to estimate the transition frequency between paramagnetism and diamagnetism.

## Introduction

Nanocomposites have variable applications in medicine and engineering, as both nanoparticles and host media are changeable to meet current needs. For example, the combination of magnetite and liquid media, called ferrofluid, has medical applications in MRI images and hyperthermia therapy^1^. Another example is the mixing of nanoparticles and polymer matrix, which can be microwave absorbers used in military and communication engineering^2^. For those cases, the critical factors are the complex permittivity and permeability of composites, as they determine the skin depth, the heating rate, and other interactions between composites and electromagnetic waves^3^.

For a particular nanoparticle and host medium, the volume fraction is a tunable parameter of the complex permittivity and permeability^4^. Therefore, we can customize one for situations by tuning the fraction. The complex permittivity and permeability also depend on frequency^5^, and hence a composite can have several applications for different frequency ranges. It is of interest to analyze the volume fraction and frequency dependence because it can help us understand physics inside the materials and can predict electromagnetic characteristics outside the measured ranges, which may reduce the cost of measurements and help us approximate the needed composite composition. In this work, we use the transmission/reflection method to extract the permittivity and permeability of epoxy/magnetite nanocomposites in the X-band and analyze their dependence on frequencies and volume fractions. Our results show the permittivity of composites has no strongly frequency-dependent in X-band as to why we focus on the frequency-dependent analysis of permeability.

Especially, our experiment revealed that the Fe_3_O_4_ nanopowder exhibits negative susceptibility in the X-band while it shows superparamagnetism at low frequencies^6–9^. A similar phenomenon was observed on La_0.7_Sr_0.3_MnO_3_ nanocomposites^10^ and FeNi_3_/C nanocapsules^11^. As the order of the negative susceptibility of those materials is much higher than 10^–4^ from the Langevin diamagnetism^12^, there must be a different mechanism accounting for the transition from paramagnetism to diamagnetism in our epoxy/magnetite (Fe_3_O_4_) nanocomposites. The permeability of superparamagnetic particles can be explained by the Debye relaxation model^13^, such as the FeAl@(Al, Fe)_2_O_3_ nanoparticles^14^, the Fe/Ag/citrate nanocomposites in 2–18 GHz^15^, and magnetite in the kHz frequency range^16^. Nevertheless, the Debye relaxation model also fails to explain the negative susceptibility, and hence an amended physics mechanism is needed.

Depending on the structure of the materials, the spins will naturally be in stable states, that is, in the directions that make the free energy reach a minimum^17–19^. For a nanoscale material, the thermal fluctuation allows the spins to transit between stable states with the volume reduction of energy barriers. The transition of states contributes to the magnetic susceptibility of superparamagnetic particles. The work of Klik et al*.*^20^ shows that the transition of states may contribute to the negative magnetic susceptibility by considering the memory effect of nanoparticles. They considered the correction of the master equation of the spins by an exponential memory kernel. Reference^20^ predicts that the material with the uniaxial anisotropy will express diamagnetism when the frequency is greater than a threshold frequency. Although this theory is not the only possible explanation and needs more experimental exploration^21,22^, it can explain the coexistence of superparamagnetism at low frequencies and diamagnetism at high frequencies as to why we adopt it to explain our results.

Compared to the previous model, our experimental samples have two differences. First, the material of interest (i.e., the magnetite) carries the cubic anisotropy with eight stable states^23,24^, while uniaxial particles in the previous model have just two stable states. Second, the previous model considers the permeability of well-aligned nanoparticles, but our nanoparticles are randomly oriented. In this work, we provide theoretical analysis for this situation and derive a formula similar to the previous model, which suggests this mechanism can also be applied on particles with variable crystal structures when particles don’t have to be well-aligned. Besides, we provide a fitting procedure so that we can extract parameters using linear regression. Not only can we apply this procedure in our cases, but also we can use it to analyze the data with other samples in a frequency range wider than 8.2–12.4 GHz.

## Results

### Permittivity

The transmission/reflection methods can characterize materials’ electromagnetic (EM) properties over a broad frequency range. The measured transmission/reflection coefficients using a network analyzer uniquely determine the complex permittivity $\varepsilon$ and the complex permeability $\mu$, when the sample thickness *d* is smaller than half of the guide wavelength ($\lambda_{{\text{g}}}$)^25^. Here, we adapt the relative permittivity $\varepsilon /\varepsilon_{0}$($= \varepsilon^{\prime} + i\varepsilon^{\prime\prime}$) and permeability $\mu /\mu_{0}$($= \mu^{\prime} + i\mu^{\prime\prime}$). $\varepsilon_{0}$ and $\mu_{0}$ denote the permittivity and the permeability of the vacuum. Four different volume fractions of epoxy/magnetite composites (0%, 6%, 12%, and 18%) are measured. Then, the effective medium theory is introduced to extract the EM properties of the nano Fe_3_O_4_ powder.

While there are several different effective medium theories, Chang et al*.* discussed three different models and concluded that the Looyenga model^26,27^ is a suitable model to fit the permittivity of the epoxy/Fe_3_O_4_ nanocomposites^6^. It reads,

1 $$ \varepsilon_{{{\text{eff}}}}^{{{1 \mathord{\left/ {\vphantom {1 3}} \right. \kern-\nulldelimiterspace} 3}}} = (1 - v_{f} )\varepsilon_{{\text{h}}}^{{{1 \mathord{\left/ {\vphantom {1 3}} \right. \kern-\nulldelimiterspace} 3}}} + v_{{\text{f}}} \varepsilon_{{\text{f}}}^{{{1 \mathord{\left/ {\vphantom {1 3}} \right. \kern-\nulldelimiterspace} 3}}} , $$

where $\varepsilon_{{{\text{eff}}}}$, $\varepsilon_{{\text{h}}}$ and $\varepsilon_{{\text{f}}}$ are the permittivity of the composites, the host medium (epoxy), and the filled material (Fe_3_O_4_), respectively. $v_{{\text{f}}}$ is the volume fraction of the filled materials.

**Figure 1:**
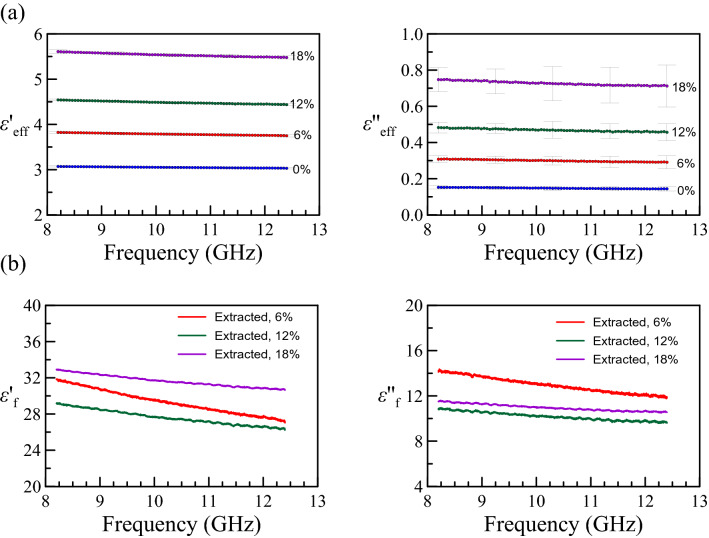
(**a**) The measured (effective) complex permittivity versus frequency, where the error bars are the standard deviation from multiple measurements. (**b**) The extracted complex permittivity of the Fe_3_O_4_ nanoparticles using the Looyenga model (Eq. 1).

**Figure 2:**
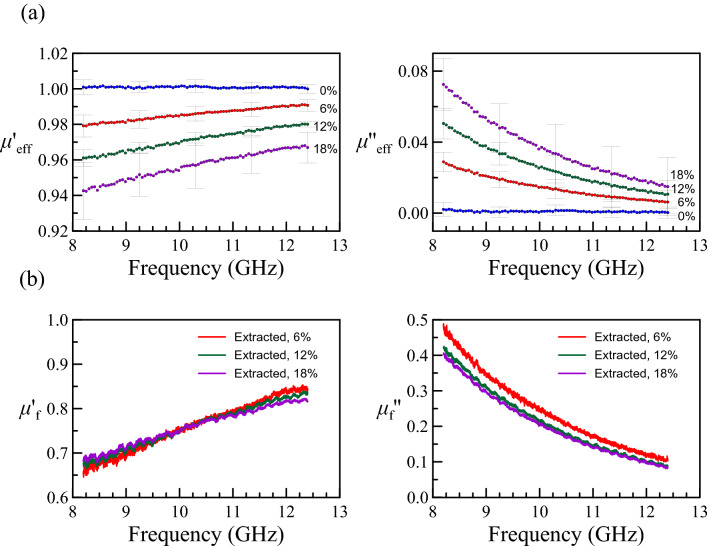
(**a**) The measured (effective) complex permeability versus frequency, where the error bars are the standard deviation from multiple measurements. (**b**) The extracted complex permeability of the Fe_3_O_4_ nanoparticles using the linearly proportional model (Eq. 2).

**Figure 3:**
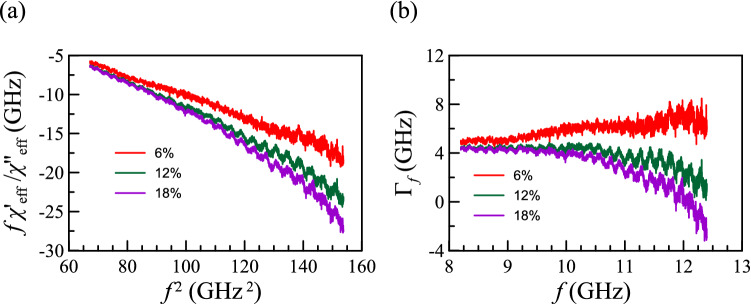
(**a**) From Eq. (14), $\Theta_{f}$ is associated with the slope of the lines, i.e., $f\chi^{\prime}_{{{\text{eff}}}} /\chi^{\prime\prime}_{{{\text{eff}}}}$ vs. $f^{2}$. (**b**) Rewriting Eq. (14), $\Gamma_{f} = f\chi^{\prime}_{{{\text{eff}}}} /\chi^{\prime\prime}_{{{\text{eff}}}} + \Theta_{f} f^{2}$. The value of $\Gamma_{f}$ is expected to be constant.

**Figure 4:**
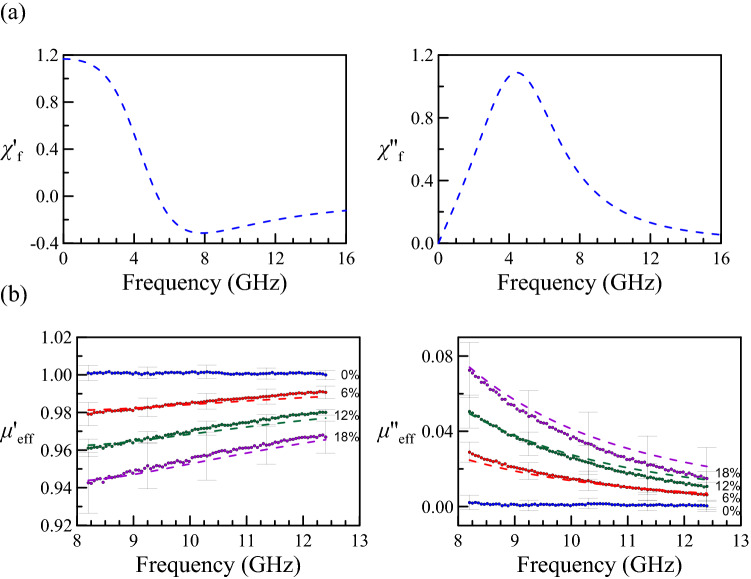
(**a**) The complex magnetic susceptibility of the nano magnetite versus frequency, extracted by fitting Eq. (12). (**b**) the comparison between experimental results (scatters) and fitting results using our model (dashed-lines).

The measured effective complex permittivity of nanocomposites with different volume fractions is presented in Fig. 1(a). In our experiment, epoxy was chosen to be the host medium. It carries relative permittivity around $3.1 + 0.1i$ and relative permeability around 1.0 with high stability within a broad frequency range. The EM properties of epoxy are confirmed by our transmission/reflection method. We calculate the corresponding $\varepsilon_{{\text{f}}}$ based on Eq. (1). The extracted $\varepsilon_{{\text{f}}}$ is shown in Fig. 1(b). The extracted complex permittivity $\varepsilon_{{\text{f}}}$ differs slightly for different volume fractions $\nu_{f}$. The difference might be attributed to the error of the measured volume fraction $v_{{\text{f}}}$. The fluctuations in Fig. 1(a, b) come from the limitation of measurement bandwidth, which is also observed in Figs. 2, 3 and 4 and is a part of systematic errors.

### Permeability

The measured permeability of nanocomposites with different volume fractions is presented in Fig. 2(a). As mentioned, the permeability of pure epoxy is near 1.0, so the magnetic susceptibility comes strictly from the existence of Fe_3_O_4_. To explain the data, we reasonably assume that the magnetic susceptibility is linearly proportional to the volume fraction of the nano Fe_3_O_4_ powder. That is,

2 $$ \chi^{\prime}_{{{\text{eff}}}} + i\chi^{\prime\prime}_{{{\text{eff}}}} = v_{{\text{f}}} (\chi^{\prime}_{{\text{f}}} + i\chi^{\prime\prime}_{{\text{f}}} ), $$

where $\chi^{\prime}_{{{\text{eff}}}} + i\chi^{\prime\prime}_{{{\text{eff}}}}$ and $\chi^{\prime}_{{\text{f}}} + i\chi^{\prime\prime}_{{\text{f}}}$ are the magnetic susceptibility of the composites and the filled material (Fe_3_O_4_), respectively. The extracted $\mu_{{\text{f}}}$ using Eq. (2) is shown in Fig. 2(b) with $\mu^{\prime}_{{\text{f}}} { + }i\mu^{\prime\prime}_{{\text{f}}} = {(}1 + \chi^{\prime}_{{\text{f}}} ) + i\chi^{\prime\prime}_{{\text{f}}}$.

For the Fe_3_O_4_ structure, the free energy of the particles will be^28^

3 $$ E = KV(\alpha^{2} \beta^{2} + \beta^{2} \gamma^{2} + \gamma^{2} \alpha^{2} ) - \mu_{0} HM_{s} V\cos \varphi , $$

where $K$ is the anisotropy constant, $V$ is the volume, $\alpha ,\;\beta ,\;\gamma$ are the direction cosines of the magnetization vector along the *x*, *y*, and *z* axes, $M_{s}$ is the saturated magnetization, $H$ is the amplitude of the applied field, and $\cos \varphi$ is the direction cosine of the magnetization vector along the *H*-field.

The magnetization vector of Fe_3_O_4_ has eight stable states, all having the form $\left| \alpha \right| = \left| \beta \right| = \left| \gamma \right|$. We, therefore, denote the stable states as the form ${[}111{]}$ or $\left[ {\overline{1}\overline{1}\overline{1}} \right]$. The numbers are used to represent the ratios of direction cosines, and we put a bar above the first, the second, or the third number if $\alpha ,\beta$ or $\gamma$ is negative. To describe the susceptibility, we define $n_{1} ,\;n_{2} ,\;n_{3} ,\;n_{4} ,\;n_{5} ,\;n_{6} ,\;n_{7}$, and $n_{8}$ as the probability of occupation of ${[}111{]},\;\left[ {\overline{1}11} \right],\left[ {1\overline{1}1} \right]\;,\left[ {11\overline{1}} \right]\;,\left[ {\overline{1}\overline{1}\overline{1}} \right]\;,\;\left[ {1\overline{1}\overline{1}} \right],\;\left[ {\overline{1}1\overline{1}} \right],$ and $\left[ {\overline{1}\overline{1}1} \right]$. Since the antiparallel occupation will cancel each other out, we can further simplify the expression by defining $m_{1} = n_{1} - n_{5} ,\;m_{2} = n_{2} - n_{6} ,\;m_{3} = n_{3} - n_{7}$, and $m_{4} = n_{4} - n_{8}$. The four variables give us enough information to get the magnetization.

We consider the case that direct transitions happen only between two adjacent states^24^. When considering the memory effect, the master equation of $${\mathbf{m}} = {[}\begin{array}{*{20}c} {m_{1} } & {m_{2} } & {m_{3} } & {m_{4} } \\ \end{array} {]}^{T}$$ is

4 $$ \Theta \ddot{{\mathbf{m}}} + {\dot{\mathbf{m}}} = {\mathbf{\mathop{f}\limits^{\leftrightarrow}  }}_{{\mathbf{0}}} {\mathbf{m}}{ + }qh\Gamma {\mathbf{v}}\;{\text{with}}\;{\mathbf{\mathop{f}\limits^{\leftrightarrow}  }}_{{\mathbf{0}}} = \Gamma \left[ {\begin{array}{*{20}c} { - 3} & 1 & 1 & 1 \\ 1 & { - 3} & { - 1} & { - 1} \\ 1 & { - 1} & { - 3} & { - 1} \\ 1 & { - 1} & { - 1} & { - 3} \\ \end{array} } \right], $$

where $\Theta$ is the memory time and $\Gamma = \Gamma_{0} \exp ( - q/12)$. A detailed derivation of how we deduce Eq. (4) from the exponential memory kernel can be found in the Supplementary information Part I. $\Gamma_{0}$ is a constant in the unit of frequency^29^, representing the transition frequency under the high-temperature limit and $q = \left| K \right|V{/(}k_{B} T{)}$. The term $\exp ( - q/12)$ comes from the energy barrier, like the Arrhenius equation. In the ground states, the particle carries free energy $KV/3$. When the magnetization vector transits from a stable state to the others, it will go through a saddle point as the form of $\left[ {110} \right]$ with energy $KV/4$ , and therefore, the energy barrier is $- KV/12$. For the second term at the right-hand side, $h = \mu_{0} M_{s} H{/(} - K)$ and ${\mathbf{v}}$ can be expressed as

5 $$ {\mathbf{v}} = \frac{1}{4}\left[ {\begin{array}{*{20}c} 3 & { - 1} & { - 1} & { - 1} \\ { - 1} & 3 & 1 & 1 \\ { - 1} & 1 & 3 & 1 \\ { - 1} & 1 & 1 & 3 \\ \end{array} } \right]\left[ {\begin{array}{*{20}c} {\cos \varphi_{1} } \\ {\cos \varphi_{2} } \\ {\cos \varphi_{3} } \\ {\cos \varphi_{4} } \\ \end{array} } \right], $$

where $\varphi_{1} ,\varphi_{2} ,\varphi_{3} ,$ and $\varphi_{4}$ are the angles between the magnetization vector and directions ${[}111{]},\;\left[ {\overline{1}11} \right],\left[ {1\overline{1}1} \right]$ and $\left[ {11\overline{1}} \right]$, which are chosen to be smaller than 180 degrees. When there is an externally applied *H*-field, the transition coefficients will change, since the energy of stable states change from $KV/3$ to $KV/3 + \mu_{0} HM_{s} V\cos \varphi$ and therefore the exponential terms of the transition rates change. Since the externally applied *H*-field is small in our experiment, we only need the influences of the first order of $h$. The second term at the right-hand side in Eq. (4) comes from the change of the transition rates.

If $h = h_{0} \exp ( - i\omega t)$ with $h_{0}$ as the amplitude and $\omega$ as the oscillating frequency, we can expect that ${\mathbf{m}}$ will oscillate with the same frequency. Therefore, we can do the Fourier transformation for the left side. The ${\mathbf{\mathop{f}\limits^{\leftrightarrow}  }}_{{\mathbf{0}}}$ has an eigenvalue $- 6\Gamma$ corresponding to the eigenvector $${[}\begin{array}{*{20}c} 1 & { - 1} & { - 1} & { - 1} \\ \end{array} {]}^{T}$$. In addition, the ${\mathbf{\mathop{f}\limits^{\leftrightarrow}  }}_{{\mathbf{0}}}$ has another eigenvalue $- 2\Gamma$ associated with three degenerate eigenvectors $$\left[ {\begin{array}{*{20}c} 1 & 1 & 0 & 0 \\ \end{array} } \right]^{T}$$, $$\left[ {\begin{array}{*{20}c} 1 & 0 & 1 & 0 \\ \end{array} } \right]^{T}$$, and $$\left[ {\begin{array}{*{20}c} 1 & 0 & 0 & 1 \\ \end{array} } \right]^{T}$$. The physical interpretation of eigenvalues and eigenvectors can be found in the Supplementary information Part II. After the matrix operation, Eq. (4) becomes

6 $$ \left[ {\begin{array}{*{20}c} {(6\Gamma - \Theta \omega^{2} - i\omega )(m_{1} - m_{2} - m_{3} - m_{4} {)}} \\ {(2\Gamma - \Theta \omega^{2} - i\omega )(m_{1} + 3m_{2} - m_{3} - m_{4} )} \\ {(2\Gamma - \Theta \omega^{2} - i\omega )(m_{1} - m_{2} + 3m_{3} - m_{4} )} \\ {(2\Gamma - \Theta \omega^{2} - i\omega )(m_{1} - m_{2} - m_{3} + 3m_{4} )} \\ \end{array} } \right] = qh\Gamma \left[ {\begin{array}{*{20}c} 1 & { - 1} & { - 1} & { - 1} \\ 1 & 3 & { - 1} & { - 1} \\ 1 & { - 1} & 3 & { - 1} \\ 1 & { - 1} & { - 1} & 3 \\ \end{array} } \right]{\mathbf{v}}. $$

To get magnetization $M$, we define the reduced magnetization $m_{r} = M/M_{s}$, and then the value of $m_{r}$ is related to $m_{i}$’s by

7 $$ m_{r} = {[}\begin{array}{*{20}c} {\cos \varphi_{1} } & {\cos \varphi_{2} } & {\cos \varphi_{3} } & {\cos \varphi_{4} } \\ \end{array} {]}\left[ {\begin{array}{*{20}c} {m_{1} } \\ {m_{2} } \\ {m_{3} } \\ {m_{4} } \\ \end{array} } \right]. $$

For simplicity, we define $1/\lambda_{1} = 6\Gamma - \Theta \omega^{2} - i\omega$ and $1/\lambda_{2} = 2\Gamma - \Theta \omega^{2} - i\omega$. Then, we get

8 $$ m_{r} = {[}\begin{array}{*{20}c} {\cos \varphi_{1} } & {\cos \varphi_{2} } & {\cos \varphi_{3} } & {\cos \varphi_{4} } \\ \end{array} {]}\left[ {\begin{array}{*{20}c} 1 & { - 1} & { - 1} & { - 1} \\ 1 & 3 & { - 1} & { - 1} \\ 1 & { - 1} & 3 & { - 1} \\ 1 & { - 1} & { - 1} & 3 \\ \end{array} } \right]^{ - 1} \left[ {\begin{array}{*{20}c} {\lambda_{1} } & 0 & 0 & 0 \\ 0 & {\lambda_{2} } & 0 & 0 \\ 0 & 0 & {\lambda_{2} } & 0 \\ 0 & 0 & 0 & {\lambda_{2} } \\ \end{array} } \right]\left[ {\begin{array}{*{20}c} 1 & { - 1} & { - 1} & { - 1} \\ 1 & 3 & { - 1} & { - 1} \\ 1 & { - 1} & 3 & { - 1} \\ 1 & { - 1} & { - 1} & 3 \\ \end{array} } \right]qh\Gamma {\mathbf{v}}. $$

Equations (6) and (8) give us $m_{r}$ for any direction of the external magnetic field. However, the orientation is randomly distributed in the experiment. Therefore what we need is the averaged $m_{r}$ for all the possible directions, denoted as $\left\langle {m_{r} } \right\rangle$. All $\left\langle {\cos \varphi_{n} m_{n} } \right\rangle$ are equal by symmetry, where *n* = 1, 2, 3, or 4. Therefore we only need to calculate $4\left\langle {\cos \varphi_{1} m_{1} } \right\rangle$.

9 $$ \left\langle {m_{r} } \right\rangle = {[}\begin{array}{*{20}c} 1 & 0 & 0 & 0 \\ \end{array} {]}\left[ {\begin{array}{*{20}c} 1 & 1 & 1 & 1 \\ { - 1} & 1 & 0 & 0 \\ { - 1} & 0 & 1 & 0 \\ { - 1} & 0 & 0 & 1 \\ \end{array} } \right]\left[ {\begin{array}{*{20}c} {\lambda_{1} } & 0 & 0 & 0 \\ 0 & {\lambda_{2} } & 0 & 0 \\ 0 & 0 & {\lambda_{2} } & 0 \\ 0 & 0 & 0 & {\lambda_{2} } \\ \end{array} } \right]\left[ {\begin{array}{*{20}c} 6 & { - 6} & { - 6} & { - 6} \\ 2 & 6 & { - 2} & { - 2} \\ 2 & { - 2} & 6 & { - 2} \\ 2 & { - 2} & { - 2} & 6 \\ \end{array} } \right]\left[ {\begin{array}{*{20}c} {\left\langle {\cos \varphi_{1} \cos \varphi_{1} } \right\rangle } \\ {\left\langle {\cos \varphi_{1} \cos \varphi_{2} } \right\rangle } \\ {\left\langle {\cos \varphi_{1} \cos \varphi_{3} } \right\rangle } \\ {\left\langle {\cos \varphi_{1} \cos \varphi_{4} } \right\rangle } \\ \end{array} } \right]\frac{qh\Gamma }{4}. $$

To find $\left\langle {\cos \varphi_{1} \cos \varphi_{n} } \right\rangle$ for all *n*’s, we can express the direction of the magnetization vector as ${\text{(sin}}\theta \sin \phi ,{\text{ sin}}\theta \cos \phi , \, \cos \theta {)}$ by setting ${(}\theta , \, \phi )$ as a coordinate of a unit sphere such that $[111]$ identities to ${{(1,1,1)} \mathord{\left/ {\vphantom {{(1,1,1)} {\sqrt 3 }}} \right. \kern-\nulldelimiterspace} {\sqrt 3 }}$. We can find $\cos \varphi_{n}$ by calculating the inner product between the magnetization vector and the direction of stable states. The average of $\cos \varphi_{1} \cos \varphi_{n}$ for all possible ${(}\theta , \, \phi )$ is what we need. The values are $\left\langle {\cos \varphi_{1} \cos \varphi_{1} } \right\rangle = 2/3$ and $\left\langle {\cos \varphi_{1} \cos \varphi_{n} } \right\rangle = 2/9$ for the rest.

10 $$ \left\langle {m_{r} } \right\rangle = {[}\begin{array}{*{20}c} {\lambda_{1} } & {\lambda_{2} } & {\lambda_{2} } & {\lambda_{2} } \\ \end{array} {]}\left[ {\begin{array}{*{20}c} 6 & { - 6} & { - 6} & { - 6} \\ 2 & 6 & { - 2} & { - 2} \\ 2 & { - 2} & 6 & { - 2} \\ 2 & { - 2} & { - 2} & 6 \\ \end{array} } \right]\left[ {\begin{array}{*{20}c} 6 \\ 2 \\ 2 \\ 2 \\ \end{array} } \right]\frac{qh\Gamma }{{36}} = \frac{4}{3}\lambda_{2} qh\Gamma . $$

Eventually, we can write the magnetization as

11 $$ M = M_{s} \left\langle {m_{r} } \right\rangle = \frac{2}{3}\frac{2\Gamma }{{2\Gamma - \Theta \omega^{2} - i\omega }}\frac{ - KV}{{k_{b} T}}\frac{{\mu_{0} M_{s}^{2} }}{ - K}H. $$

Our calculation shows that the only decay rate that contributes to the magnetization is $2\Gamma$, and therefore the mathematical form looks just like that for uniaxial particles. This result is not limited to the randomly oriented case as shown in the Supplementary information Part II.

The magnetic susceptibility of Fe_3_O_4_ will be

12 $$ \chi^{\prime}_{f} + i\chi^{\prime\prime}_{f} = \frac{M}{H} = \frac{{\mu_{0} M_{s}^{2} }}{ - K}\frac{m}{h} = \frac{{2\mu_{0} M_{s}^{2} q}}{3\left| K \right|}\frac{{\Gamma_{f} }}{{\Gamma_{f} - \Theta_{f} f^{2} - if}}, $$

where we use the frequency $f$ to replace $\omega$, $\Gamma_{f} = 4\pi \Gamma$, and $\Theta_{f} = \Theta {/}2\pi$.

The three unknown variables are $2\mu_{0} M_{s}^{2} q{/(}3\left| K \right|{)}$, $\Gamma_{f}$, and $\Theta_{f}$. We have two curves: the real permeability and the imaginary permeability, which give us two conditions. Since we have two conditions but three variables, it will be beneficial if we can obtain another constraint. From Eqs. (2) and (12), the real part and imaginary part of magnetic susceptibility of composites will be

13 $$ \left\{ {\begin{array}{*{20}c} {\chi^{\prime}_{{{\text{eff}}}} = \frac{{2\mu_{0} M_{s}^{2} V}}{{3k_{B} T}}v_{f} \frac{{\Gamma_{f} (\Gamma_{f} - \Theta_{f} f^{2} )}}{{f^{2} + (\Gamma_{f} - \Theta_{f} f^{2} )^{2} }}} \\ {\chi^{\prime\prime}_{{{\text{eff}}}} = \frac{{2\mu_{0} M_{s}^{2} V}}{{3k_{B} T}}v_{f} \frac{{f\Gamma_{f} }}{{f^{2} + (\Gamma_{f} - \Theta_{f} f^{2} )^{2} }}} \\ \end{array} .} \right. $$

The two equations merge to

14 $$ f\frac{{\chi^{\prime}_{{{\text{eff}}}} }}{{\chi^{\prime\prime}_{{{\text{eff}}}} }} = \Gamma_{f} - \Theta_{f} f^{2} . $$

Since the left-hand side can be directly computed from the experimental data, we can get $\Theta_{f}$ by the slope of the left-hand side term versus $f^{2}$, as shown in Fig. 3(a). The $\Theta_{f}$ should be a positive quantity, and we indeed found a decreased line in Fig. 3(a). After getting the $\Theta_{f}$, one can get $\Gamma_{f}$ by putting $\Theta_{f} f^{2}$ to the left side of the Eq. (14) as shown in Fig. 3(b). Notably, if one checks Fig. 2(a) carefully, the real and imaginary susceptibilities ($\chi^{\prime}_{{{\text{eff}}}}$ and $\chi^{\prime\prime}_{{{\text{eff}}}}$) above 10 GHz are very close to zero. Since $\chi^{\prime\prime}_{{{\text{eff}}}}$ is in the dominator of the left-hand side of Eq. (14), the very small value of $\chi^{\prime\prime}_{{{\text{eff}}}}$ will enlarge the uncertainty. Therefore, the regions to the left of the dashed lines in Fig. 3(a, b) are more reliable and those are the region of interest. The consistency between Eq. (14) and the measured data suggests the memory model with cubic anisotropic materials works well. After finding $\Theta_{f}$ and $\Gamma_{f}$, we can then determine the remaining coefficient in Eq. (12), i.e., $2\mu_{0} M_{s}^{2} q{/(} - 3K)$. The fitting results of the three variables are listed in Table 1. Note that when each volume fraction can provide a fitting result, the fitting results show high consistency on the value of $\Theta_{f}$ but less consistency on the value of $\Gamma_{f}$, which is because we use linear regression and it is more sensitive to the slope than to the intercept.

**Table 1:** The corresponding parameters in Eq. (12) deduced by the fitting.

$\frac{{2\mu_{0} M_{s}^{2} V}}{{3k{}_{B}T}}$	(GHz)	$\Theta_{f}$ (ns)
$1.167$	$4.55$	$0.16$

Figure 4(a) shows the magnetic susceptibility of nano-magnetite based on Eq. (12) and Table 1. As shown in Fig. 4(b), the fitting curves basically lie in the error bars, indicating the model is appropriate for the frequency-induced complex magnetic susceptibility of nano-particles. Besides, using Eq. (12), we can estimate the permeability outside the range of the X-band. As an example, from the form of $\chi^{\prime}_{{\text{f}}}$, one can expect that the transition between paramagnetism and diamagnetism happens at $\chi_{{\mathrm{f}}}^{\prime}$. When the frequency is higher than $f_{t}$, the real part of the magnetic susceptibility becomes negative. This result is similar to the frequency response of an RLC circuit as Eq. (4) can be analogous to an RLC circuit, which is explained in the Supplementary information Part III. $f_{t}$ will be 5.33 GHz using the parameters in Table 1. The transition frequency $f_{t}$ agrees with the experimental observation that the epoxy/magnetite nanocomposite expresses superparamagnetism at 2.45 GHz^6^ and exhibits diamagnetism in the X-band (8–12 GHz, i.e., this work).

Although our experiment didn’t cover the predicted transition frequency, the Supplementary Information Part IV shows reasonably good fitting results of previously published data based on the proposed model^10,11,30^. We believe those results suggest the model is reliable as the fitting curves can catch some points of interest, including the transition frequency.

## Discussion

We have proposed a memory model with thermal agitation of the ferromagnetic nanoparticle. The superparamagnetism is from the thermal fluctuations between stable states, while the diamagnetism originates from the out of phase response with the external driven field (Supplement Part III). We just show that the memory effect yields the correct frequency response of the susceptibility for cubic anisotropic materials where the magnetic susceptibility changes from positive at low frequency to negative at high frequency. The memory effect usually has different origins. In the discussion from Klik et al.^20^, they show the memory effect is possibly from the thermal bath, however, other interactions between environments and considered relaxing magnetic nanoparticles, e.g., viscosity between nanoparticles and system can be also the origins for memory effect^31^.

To demonstrate the importance of the value of $\Theta$, we examine the minimum of the real susceptibility in Eq. (12):

15 $$ \chi^{\prime}_{\min } = - \frac{{2\mu_{0} M_{s}^{2} q}}{3\left| K \right|}\left( {\frac{2\Gamma \Theta }{{2\sqrt {2\Gamma \Theta } + 1}}} \right), $$

when $\omega_{\min } = \sqrt {\left( {{{2\Gamma } \mathord{\left/ {\vphantom {{2\Gamma } \Theta }} \right. \kern-\nulldelimiterspace} \Theta }} \right) + {{\sqrt {{{2\Gamma } \mathord{\left/ {\vphantom {{2\Gamma } \Theta }} \right. \kern-\nulldelimiterspace} \Theta }} } \mathord{\left/ {\vphantom {{\sqrt {{{2\Gamma } \mathord{\left/ {\vphantom {{2\Gamma } \Theta }} \right. \kern-\nulldelimiterspace} \Theta }} } \Theta }} \right. \kern-\nulldelimiterspace} \Theta }}$. Note that the value of $2\Gamma \Theta$, i.e., the ratio of the memory time ($\Theta$) and the relaxation time ${1 \mathord{\left/ {\vphantom {1 {2\Gamma }}} \right. \kern-\nulldelimiterspace} {2\Gamma }}$, affects the amplitude of the minimum. Under the limit $2\Gamma \Theta \to 0$, $\chi^{\prime}_{\min } \to 0$ with the corresponding frequency $\omega \to \infty$, which implies that the memory effect is feeble and the susceptibility will comply with the Debye relaxation formula.

Another extreme case is $2\Gamma \Theta \to \infty$. In this case, $\chi^{\prime}_{\min }$ is negative, associated with extremely large $\chi^{\prime\prime}$. However, the corresponding frequency $\omega \to 0$, and once the order of frequency is larger than that of the corresponding frequency $\omega_{\min }$, $\chi^{\prime}$ will be quite close to 0. The reason we can observe the diamagnetism phenomenon in X-band is that the memory time scale and relaxation time scale are comparable, and hence diamagnetism becomes observable as $\omega \approx \sqrt {\Gamma /\Theta }$.

The diamagnetism expels the magnetic fields within a material. A superconductor is a perfect diamagnetic material with *χ*′ =  − 1, which results in no internal magnetic field. This study explored the memory effect, which produced a strong diamagnetism for nanocomposites at room temperature. Such a new mechanism is called frequency-induced diamagnetism. The frequency-induced diamagnetism significantly reduces the ac magnetic field without affecting the ac electric field. It is fundamentally different from the Meissner effect. The limit of the negative susceptibility is yet to be uncovered. Besides, the adjustable magneto-dielectric properties of the composite materials can be used for multilayer antireflection coating or the stealth aircraft/warship. It deserves further theoretical and experimental studies.

## Methods

### Transmission/reflection method

To conduct the transmission/reflection measurement, we sandwich the sample between two adaptors connecting to a performance network analyzer (PNA). The experiment is performed using the standard WR90 waveguide with inner dimensions of 0.9 in by 0.4 in. The two adaptors serve as the two ports, which convert the WR90 waveguide to the 2.4 SMA (SubMiniature version A) coaxial cables. The two ports are calibrated before measurement. The sample is filled in a uniform and hollowed WR90 waveguide. The measured transmission/reflection coefficients are then used to extract the complex permittivity/permeability of samples. A more detailed explanation and the display of transmission/reflection can be found in the Supplementary information Part V.

### Preparation of the nanocomposite samples

**Figure 5:**
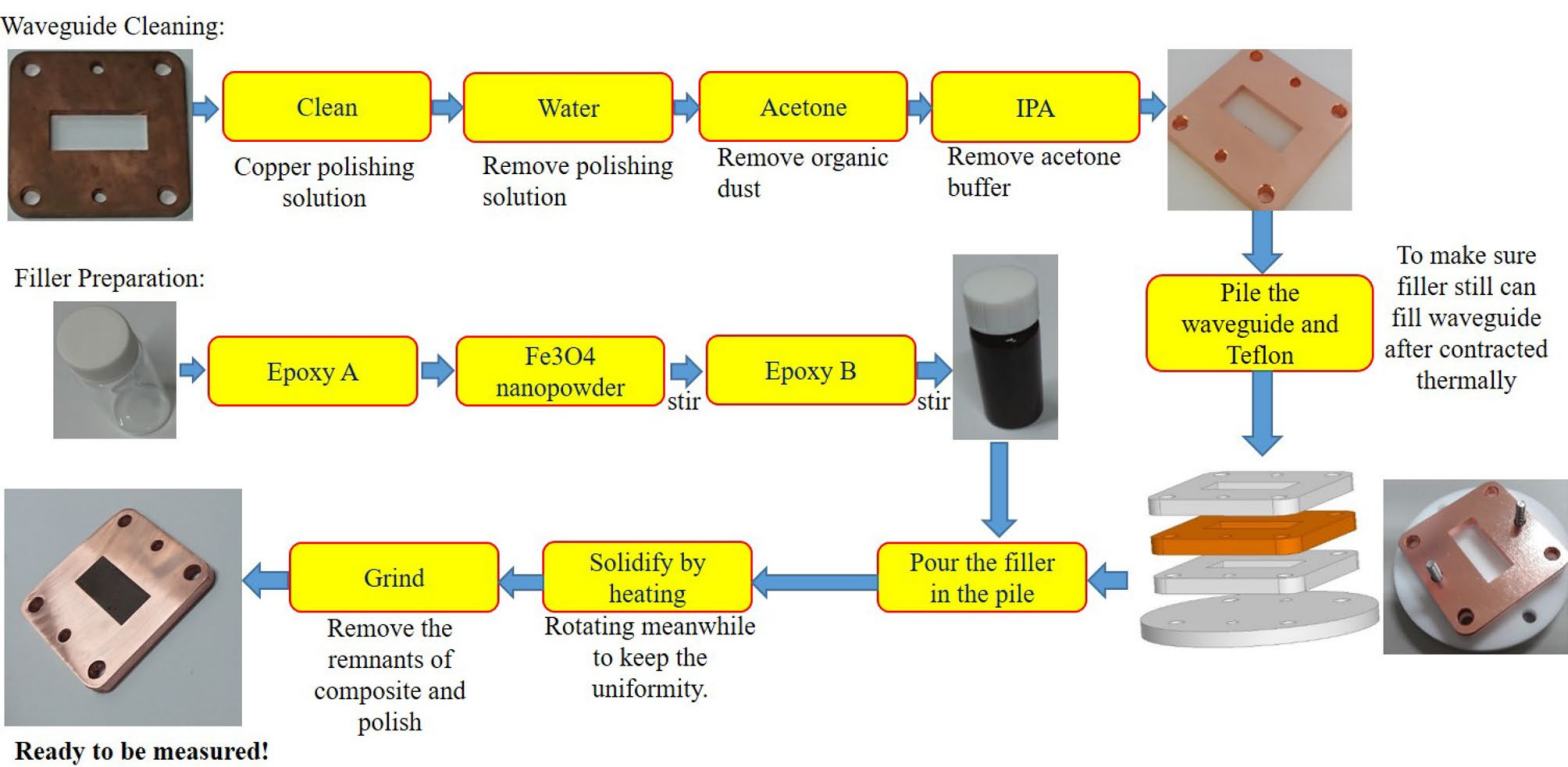
The sample preparing procedures: the waveguide is cleaned through four steps. The filler is prepared by mixing epoxy A, nano-magnetite powder, and epoxy B. Then, the mixture is poured into the mold consisting of the waveguide and other fixtures. The mold is dried for days, and the sample surface is ground and polished.

Figure 5 illustrates the sample preparing procedure:

1. Cleaning the waveguide
  The standard WR90 waveguide made of copper should be oxidation-free and clean inside with low surface roughness. The purpose of cleaning the waveguide is to avoid the conductor loss and to ensure that the loss comes strictly from the nanocomposite.
  We first soaked the waveguide into the copper polishing solution to remove the oxide. Then, the waveguides were immersed in the distilled water to remove the remnant acid. After that, we bathed the waveguide in acetone to remove the organic dust and the water. Finally, we soaked it into isopropyl alcohol (IPA) to remove acetone buffer. An ultrasonic cleaning machine is used during the process. Eventually, we obtain a clean waveguide, as shown in Fig. 5.
2. Preparation of filler:
  We use commercially available Fe_3_O_4_ as our nanopowder. The first grain size of the Fe_3_O_4_ powder was 5–20 nm in diameter. The grains of the Fe_3_O_4_ powder were made using a wet method with purity > 98% and a density of 5 g/cm^3^.
  Epoxy resin (epoxy A) mixing with hardener (epoxy B) will become very sticky and soon form a thermosetting polymer. The density of solidified epoxy is about 1.2 g/cm^3^. To achieve the nanocomposite with a uniform distribution, we first poured 3 g epoxy A into a small jar, and then placed the nano Fe_3_O_4_ powder with an appropriate amount into it. Then, we stirred the liquid in the jar to make them mix. We would heat the jar to make Fe_3_O_4_ dissolve more easily. Finally, we put 3 g epoxy B into it and kept stirring until the mixture looks evenly distributed.
3. Filling the waveguide
  After cleaning the waveguide and preparing the filler, we needed to fill the waveguide with the filler. During the solidifying process, the volume of the epoxy will shrink and may result in an air gap between the sample and the waveguide. To solve this problem, we piled the waveguide and Teflon and then filled the whole pile. After filling the pile, we heated it so that epoxy can solidify. When it solidified several hours later, we removed Teflon, and one would find that the thickness of the solidified filler exceeds the waveguide length. We used a grinding machine to remove the remnants of filler and polish the surface of the waveguide.

**Figure 6:**
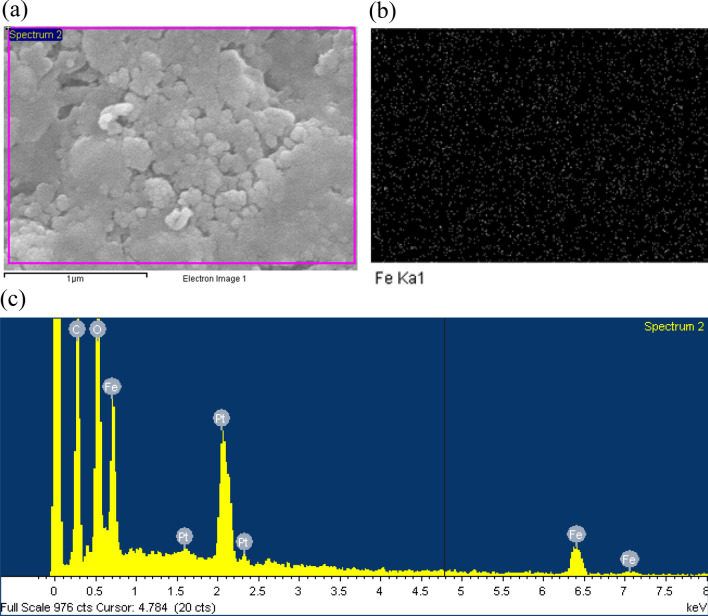
(**a**) The SEM image, (**b**) the corresponding distribution of iron through the Fe Ka X-ray microanalysis, and (**c**) the ingredient analysis using the X-ray emission spectrum.

After completing the sample preparation procedures, we then went through a scanning electron microscope (SEM) measurement to check the uniformity of the nanocomposite. The results are shown in Fig. 6, which assures the uniformity of samples. The SEM image of the sample is shown in Fig. 6(a). The Fe_3_O_4_ particles are rich in Fe. Using the Fe Ka X-ray microanalysis, we can analyze the distribution of iron, as in Fig. 6(b). Figure 6(c) shows the ingredient analysis using the X-ray emission spectrum. Fabrication of nanocomposites may accompany many problems, such as poor particle dispersion, nanovoids generation, and imperfect bonding with an epoxy matrix. The latter two problems are not critical and the theoretical model does not take them into account. However, the poor particle dispersion may lead to particle aggregation, forming huge magnetite clusters.

We fabricated multiple samples for a value of the volumetric fraction on different dates. The measured results all have a similar trend, which showed good repeatability. Furthermore, the distribution of the Fe_3_O_4_ particles is shown in Fig. 6(b) using the Fe K*α* X-ray microanalysis. The image shows that the specimen has a good uniformity in μm-scale. The magnetite particles distribute uniformly like stars in the sky. The microwave of 10 GHz has the wavelength *λ* in cm-scale and the grain sizes of the magnetite *d* are in nm scale, which is ideal for the long-wavelength approximation (*d*/*λ*«1). Ref.^6^ also showed that the particle dispersion seems not very critical at 2.45 GHz. This might explain the repeatability of our experiment and why the particle dispersion doesn’t seriously affect the experimental results.

This work provides a complete derivation and a detailed explanation for the frequency-induced negative magnetic susceptibility, which is validated in the experiment. The imperfection of specimens may affect the accuracy of the fitting results, but it doesn’t change the correctness and generality of our work. Therefore, the authors conclude that the particle dispersion is important, in general, but it is good enough for the present experiment.

## Supplementary Information

Supplementary Information

## References

[1] Kozissnik B, Bohorquez AC, Dobson J, Rinaldi C (2013). Magnetic fluid hyperthermia: advances, challenges, and opportunity. Int. J. Hhyperther..

[2] Ting TH, Yu RP, Jau YN (2011). Synthesis and microwave absorption characteristics of polyaniline/NiZn ferrite composites in 2–40 GHz. Mater. Chem. Phys..

[3] Pozar DM (2011). Microwave Engineering.

[4] Todd MG, Shi FG (2005). Complex permittivity of composite systems: a comprehensive interphase approach. IEEE Trans. Dielectr. Electr. Insul..

[5] Griffiths DJ (2012). Introduction to Electrodynamics.

[6] Chang TH, Tsai CH, Wong WS, Chen YR, Chao HW (2017). Permeability measurement and control for epoxy composites. Appl. Phys. Lett..

[7] Yang MD (2018). Magnetic interaction of multifunctional core–shell nanoparticles for highly effective theranostics. Adv. Mater..

[8] Xiang Z (2020). Enhancing the low-frequency induction heating effect of magnetic composites for medical applications. Polymers.

[9] Han R, Li W, Pan W, Zhu M, Zhou D, Li FS (2014). 1D magnetic materials of Fe_3_O_4_ and Fe with high performance of microwave absorption fabricated by electrospinning method. Sci. Rep..

[10] Yang, R. B., Tsay, C. Y., Liang, W. F., and Lin, C. K. Microwave absorbing properties of La_0.7_Sr_0.3_MnO_3_ composites with negative magnetic susceptibility. *J. Appl. Phys*. **107**, 09A523 (2010).

[11] Liu XG, Ou ZQ, Geng DY, Han Z, Xie ZG, Zhang ZD (2009). Enhanced natural resonance and attenuation properties in superparamagnetic graphite-coated FeNi_3_ nanocapsules. J. Phys. D: Appl. Phys..

[12] Kittel C (2005). Introduction to Solid State Physics.

[13] Trisnanto SB, Kitamoto Y (2014). Field-dependent Brownian relaxation dynamics of a superparamagnetic clustered-particle suspension. Phys. Rev. E.

[14] Liu T, Pang Y, Kikuchi H, Kamada Y, Takahashi S (2015). Superparamagnetic property and high microwave absorption performance of FeAl@(Al, Fe)_2_O_3_ nanoparticles induced by surface oxidation. J. Mater. Chem. C.

[15] Vural M, Crowgey B, Kempel LC, Kofinas P (2014). Nanostructured flexible magneto-dielectrics for radio frequency applications. J. Mater. Chem. C.

[16] Torres TE, Lima E, Calatayud MP (2019). The relevance of Brownian relaxation as power absorption mechanism in Magnetic Hyperthermia. Sci. Rep..

[17] Ryan FM, Pugh EW, Smoluchowski R (1959). Superparamagnetism, nonrandomness, and irradiation effects in Cu–Ni alloys. Phys. Rev..

[18] Bean C, Livingston JD (1959). Superparamagnetism. J. Appl. Phys..

[19] Néel L (1949). Théorie du traînage magnétique des ferromagnétiques en grains fins avec applications aux terres cuites. Ann. Géophys..

[20] Klik I, McHugh J, Chantrell RW, Chang CR (2018). Debye formulas for a relaxing system with memory. Sci. Rep..

[21] Luo H, Gong R, Wang X, Song K, Zhu C, Wang L (2016). Synthesis and excellent microwave absorption properties of reduced graphene oxide/FeNi_3_/Fe_3_O_4_ composite. New J. Chem..

[22] Yang Y, Yang Y, Xiao W, Neo CP, Ding J (2015). Shape-dependent microwave permeability of Fe_3_O_4_ nanoparticles: a combined experimental and theoretical study. Nanotechnology.

[23] Siratori K, Kino Y (1980). A note on the magnetic anisotropy of Fe_3_O_4_. J. Magn. Magn. Mater..

[24] Coffey WT, Kalmykov YP (2012). Thermal fluctuations of magnetic nanoparticles: fifty years after Brown. J. Appl. Phys..

[25] Su SC, Chang TH (2020). Manipulating the permittivities and permeabilities of epoxy/silver nanocomposites over a wide bandwidth. Appl. Phys. Lett..

[26] Landau LD, Lifshitz EM (1960). Electrodynamics of Continuous Media.

[27] Looyenga H (1965). Dielectric constants of heterogeneous mixtures. Physica.

[28] Kalmykov YP (2000). Longitudinal dynamic susceptibility and relaxation time of superparamagnetic particles with cubic anisotropy: effect of a biasing magnetic field. Phys. Rev. B.

[29] Klik I, Chang CR, Huang HL (1993). Field-dependent prefactor of the thermal relaxation rate in single-domain magnetic particles. Phys. Rev. B.

[30] Liu JR, Itoh M, Horikawa T, Machida KI, Sugimoto S, Maeda T (2005). Gigahertz range electromagnetic wave absorbers made of amorphous-carbon-based magnetic nanocomposites. J. Appl. Phys..

[31] Phuoc TX, Massoudi M (2009). Experimental observations of the effects of shear rates and particle concentration on the viscosity of Fe_2_O_3_-deionized water nanofluids. Int. J. Therm. Sci..

