# Supplementary material for: Frequency-induced negative magnetic susceptibility in epoxy/magnetite nanocomposites
Source: Sci Rep. 2021 Feb 8;11:3288. doi: 10.1038/s41598-021-82590-w (PMC7870892; doi:10.1038/s41598-021-82590-w)
Supplement: Supplementary file 1 — Supplementary Information [file 41598_2021_82590_MOESM1_ESM.pdf]

Supplementary Information

Frequency-induced Negative Magnetic Susceptibility in Epoxy/Magnetite Nanocomposites

Che-Hao Chang[1], Shih-Chieh Su[2], Tsun-Hsu Chang[1, 2*], & Ching-Ray Chang[3]**

[1]Interdisciplinary Program of Sciences, National Tsing Hua University, Hsinchu, Taiwan

[2]Department of Physics, National Tsing Hua University, Hsinchu, Taiwan

[3]Department of Physics, National Taiwan University, Taipei, Taiwan

Part I. Differential form of the exponential memory kernel

In general, the correction of the master equation induced by the memory effect can be expressed as

$$\dot{\mathbf{m}}(t) = \int_{-\infty}^{t} K(t-\tau)(\overleftrightarrow{\mathbf{f}}_{\mathbf{0}}\mathbf{m}+qh\Gamma\mathbf{v})(\tau)d\tau, \tag{S1}$$

where K is the memory kernel[S1], representing how the history of the system influence the current system. Here, we set $K = \exp(-t/\Theta)/\Theta$ in the sense that the system has a finite memory time Θ. Given this memory kernel, we can take the derivatives with respect to time on both sides of Eq. (S1). Then, we will get

$$\ddot{\mathbf{m}}(t) = K(0)(\overleftrightarrow{\mathbf{f}}_{\mathbf{0}}\mathbf{m}+qh\Gamma\mathbf{v})(t) + \int_{-\infty}^{t} \dot{K}(t-\tau)(\overleftrightarrow{\mathbf{f}}_{\mathbf{0}}\mathbf{m}+qh\Gamma\mathbf{v})(\tau)d\tau. \tag{S2}$$

Since $K(0) = 1/\Theta$ and $\dot{K}(t-\tau) = -K(t-\tau)/\Theta$, Combining with Eq. (S1), we can furthermore rewrite Eq. (S2) as

$$\ddot{\mathbf{m}}(t) = \frac{1}{\Theta}((\overleftrightarrow{\mathbf{f}}_{\mathbf{0}}\mathbf{m}+qh\Gamma\mathbf{v})(t) - \dot{\mathbf{m}}(t)). \tag{S3}$$

and one can easily rewrite it as the form in Eq. (4).

Part II. Interpretation of eigenvalues of $\overleftrightarrow{\mathbf{f}}_{\mathbf{0}}$

Note the diagonalization of $\overleftrightarrow{\mathbf{f}}_{\mathbf{0}}$ tells us that $m_1' = m_1 - (m_2 + m_3 + m_4)$ is corresponding to the eigenvalue -6Γ, while $m_2' = m_1 + 3m_2 - m_3 - m_4$, $m_3' = m_1 - m_2 + 3m_3 - m_4$, and $m_4' = m_1 - m_2 - m_3 + 3m_4$ are corresponding to the eigenvalue -2Γ. The master equation of m_n''s when $h = 0$ and $\Theta = 0$ is

$$\begin{bmatrix} \dot{m}_1' \\ \dot{m}_2' \\ \dot{m}_3' \\ \dot{m}_4' \end{bmatrix} = \begin{bmatrix} -6\Gamma & 0 & 0 & 0 \\ 0 & -2\Gamma & 0 & 0 \\ 0 & 0 & -2\Gamma & 0 \\ 0 & 0 & 0 & -2\Gamma \end{bmatrix} \begin{bmatrix} m_1' \\ m_2' \\ m_3' \\ m_4' \end{bmatrix}. \tag{S4}$$

Therefore, while m_1' has a relaxation time $1/(6\Gamma)$, m_2', m_3' and m_4' has a relaxation time $1/(2\Gamma)$.

The difference can be understood by the following interpretation. If we understand m_n as the reduced magnetization along the corresponding direction, then what m_1' represents is the difference between the reduced magnetization along $[111]$ and the sum of the reduced magnetizations along three adjacent directions. On the other hand, m_2' can be rewritten as $m_2' = (m_2 - (-m_1)) + (m_2 - m_3) + (m_2 - m_4)$, where $-m_1$ represents the reduced magnetizations along $[\bar{1}\bar{1}\bar{1}]$. Therefore what m_2' represents is the sum of the difference between the reduced magnetization along $[\bar{1}11]$ and three directions adjacent to the opposite direction. m_3' and m_4' just replace $[\bar{1}11]$ by $[1\bar{1}1]$ and $[11\bar{1}]$. The $\overleftrightarrow{\mathbf{f}}_{\mathbf{0}}$ matrix allows only direct transitions between adjacent states. For non-adjacent states, the transitions are indirect, and therefore it takes more time for the non-adjacent states' transitions than for the adjacent transitions. That why m_1' has a shorter relaxation time than the others, since the difference between adjacent states disappears faster than that between non-adjacent states.

With the corresponding relaxation time, it is natural that the frequency response of m_1' will be the form $m_1' = C_1(\mathbf{H})qh\Gamma/(6\Gamma - \Theta\omega^2 + i\omega)$ and the frequency response of other m_n' will be the form $m_n' = C_n(\mathbf{H})qh\Gamma/(2\Gamma - \Theta\omega^2 + i\omega)$. To see why we only see 2Γ dependence in the total m_r, note Eq. (8) can also be written as

$$m_r = \frac{1}{4}[\cos\varphi_1 \quad \cos\varphi_2 \quad \cos\varphi_3 \quad \cos\varphi_4]\begin{bmatrix} 1 & 1 & 1 & 1 \\ -1 & 1 & 0 & 0 \\ -1 & 0 & 1 & 0 \\ -1 & 0 & 0 & 1 \end{bmatrix}\begin{bmatrix} m_1' \\ m_2' \\ m_3' \\ m_4' \end{bmatrix}. \tag{S5}$$

Hence, m_1' contributes to m_r as the form $(\cos\varphi_1 - \cos\varphi_2 - \cos\varphi_3 - \cos\varphi_4)m_1'/4$. However, $\cos\varphi_n$'s are related to each other by $\cos\varphi_1 = \cos\varphi_2 + \cos\varphi_3 + \cos\varphi_4$. As a result, m_1' can not contribute to m_r in the sense that the value of m_r won't change before and after the corresponding transition. For example, when the applied field is along $[111]$, $\cos\varphi_1 = 1$ and $\cos\varphi_n = 1/3$ for the rest. Hence, if $m_{1,0} \to m_{1,0} - \Delta m$ by the corresponding transition, $m_{n,0} \to m_{n,0} + \Delta m$ for the rest and $m_r = m_{1,0} + (m_{2,0} + m_{3,0} + m_{4,0})/3$ before and after the transition.

Part III. Understanding the memory effect using the RLC current

An RLC current satisfies

$$\frac{L}{R}\ddot{q} + \dot{q} + \frac{1}{RC}q = \frac{V}{R}, \tag{S6}$$

where L is the inductance; R is the resistor; C is the capacitance; V is the applied voltage, and q is the charge on the capacitor. As the form looks like Eq. (4), we may explain Eq. (4) by each term of the RLC current.

For $-\overleftrightarrow{\mathbf{f}}_{\mathbf{0}}\mathbf{m}$, when the applied voltage is DC, then $\mathbf{m}$ will converge to $-\overleftrightarrow{\mathbf{f}}_{\mathbf{0}}\mathbf{m} = qh\Gamma\mathbf{v}$ just as q will converge to $q = VC$. Therefore $-\overleftrightarrow{\mathbf{f}}_{\mathbf{0}}$ acts as the character of the capacitance. In addition, as the supplementary information shows, the eigenvalues of $-\overleftrightarrow{\mathbf{f}}_{\mathbf{0}}$ represent relaxation time, just like RC.

For $\Theta\ddot{\mathbf{m}}$, it acts like L/R. To see why the memory effect has such a meaning, note an inductor acts as the role that it keeps current from changing. One can check the form of the memory effect in Eq. (S1) again:

$$\dot{\mathbf{m}}(t)=\int_{-\infty}^{t}K(t-\tau)(\ddot{\mathbf{f}}_{\mathbf{0}}\mathbf{m}+qh\Gamma\mathbf{v})(\tau)d\tau. \tag{S7}$$

This equation tells us what the memory kernel does is to memorize the previous value of $\dot{\mathbf{m}}$ and try to keep $\dot{\mathbf{m}}$ to be the same, just as the inductor tries to keep $\dot{q}$ to be the same.

Part IV. Additional fitting results using the proposed model

Here we offer three different fittings of previously published results using the proposed model. Each of them gives the permeability of nanocomposites in 2 to 18 GHz and therefore can help us examine the performance over a broader frequency range.

In the following fitting procedure, we will adopt the formula form

$$\begin{cases}\chi'_{\text{eff}}=C\dfrac{\Gamma_f(\Gamma_f-\Theta_f f^2)}{f^2+(\Gamma_f-\Theta_f f^2)^2}\\[2ex] \chi''_{\text{eff}}=C\dfrac{f\Gamma_f}{f^2+(\Gamma_f-\Theta_f f^2)^2}\end{cases}, \tag{S8}$$

where Γ_f and Θ_f carries similar meanings as in Eq. (13) but we replace the coefficient by a constant C as the discussed materials may not have cubic anisotropy and the exact expansion of C may need furthermore calculations. Again, Eq. (14) can be deduced from Eq. (S8) and we may use linear regression to get Θ_f and Γ_f as they are corresponding to the slope and the intercept in Eq. (14) respectively. After that C can be correspondingly determined by fitting the equation

$$\chi''_{\text{eff}}=C\left(f\Gamma_f\big/(f^2+(\Gamma_f-\Theta_f f^2)^2)\right). \tag{S9}$$

Example I. [SRef. 2]

The first one is an experimental result of superparamagnetic $FeNi_3$ nanocapsules[S2]. We first pick up some points of real and imaginary permeability showing on the paper, use interpolation to make them become curves, and then implement the fitting procedure. Fig. S1(a) shows the result of linear regression when Fig. S1(b) and Fig. S1(c) show the comparison between experimental data and fitting curves. As shown in Fig. S1(a), the slope is negative, agreeing with our expectations.

Note that the linear regression has R-square above 0.9. Aside from the sign of slope, it's another important criterion to judge if it's a suitable model to fit the curves. For example, the electromagnetic characteristics of the mixing of $FeNi_3$ and carbon are also measured in this paper. However, the R-square of the linear regression is only about 0.8 and hence the fitting result makes no sense.

As one can see, this model successfully catches the change tendency, including the zero and minimum of the real susceptibility. Both the model curve and experimental data of the imaginary part and susceptibility have a peak, although the positions don't perfectly agree with each other. Still, the fitting curve shows this model can explain data in a broad frequency range but not just in the X-band.

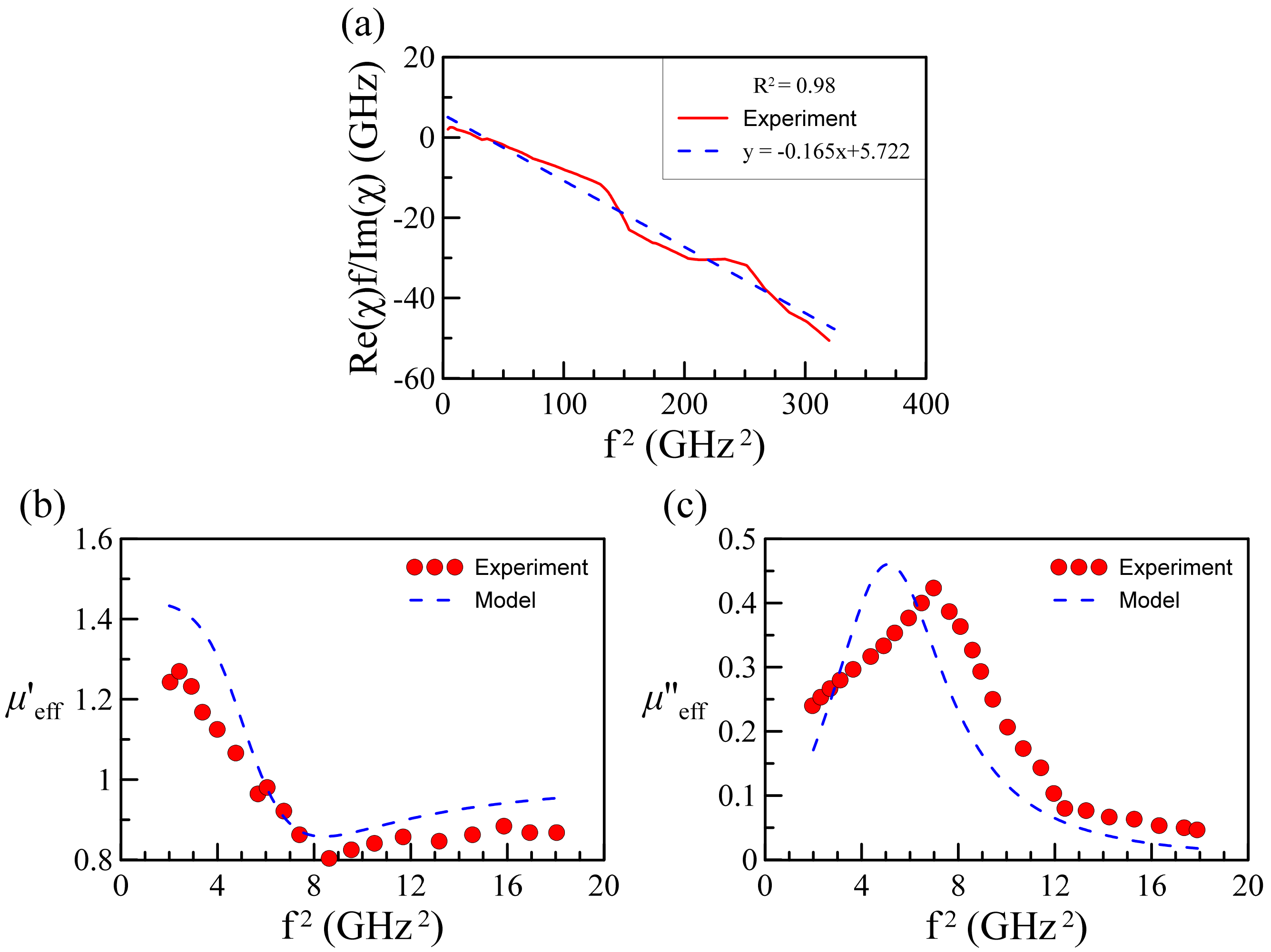

Figure. S2. (a) The linear regression using Eq. (14). (b) the real part and (c) the imaginary part of the magnetic susceptibility of the $FeNi_3$/paraffin nanocomposite from experimental data of SRef. 2 and model fitting, where we use $C = 0.4427, \Gamma_f = 5.722$ GHz and $\Theta_f = 0.165$ ns.

Example II. [SRef. 3]

The next example is a combination of Fe and amorphous carbon denoted as Fe/C(a)[S3]. Again, Fig. S2(a) shows the result of linear regression when Fig. S2(b) and (c) show the comparison between experimental data and fitting curves. We pick it up as our second example not only because its tendency agrees with our model but also because it represents an interesting limit $\Theta_f \Gamma_f \ll 1$, i.e., weak memory effect. As one can see, while the peak of the imaginary part of susceptibility is roughly at 6 GHz as the first example, the real part of susceptibility is always positive below 12 GHz. This is associated with the fact that the weaker the memory effect, the higher the transition frequency because of $f_t \propto 1/\sqrt{\Theta_f}$.

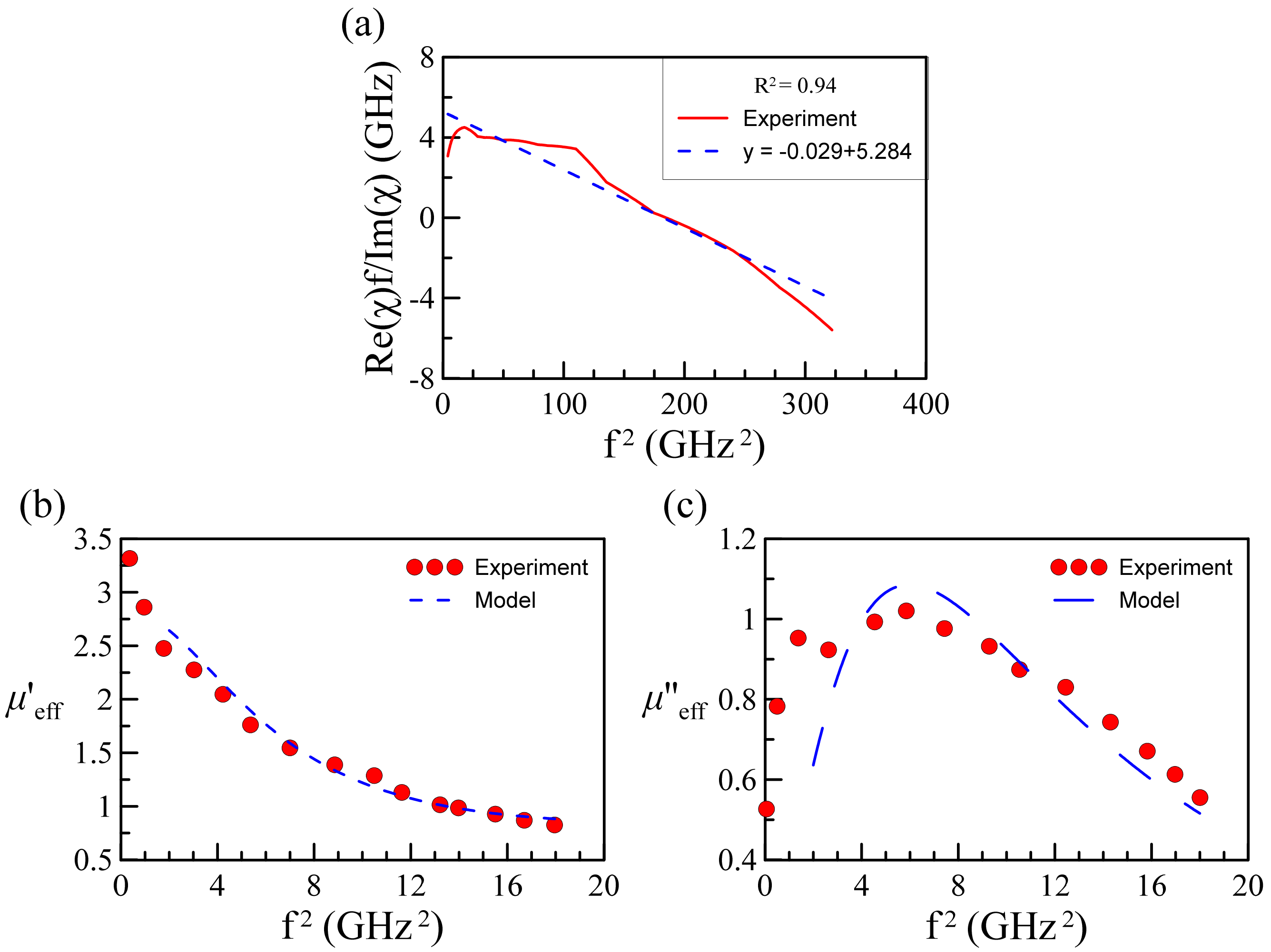

Figure. S2. (a) The linear regression using Eq. (14). (b) the real part and (c) the imaginary part of the magnetic susceptibility of the $FeNi_3$/C(a)/Epoxy nanocomposite from experimental data of SRef. 3 and model fitting, where we use $C = 1.848$, $\Gamma_f = 5.284$ GHz, and $\Theta_f = 0.029$ ns.

Example III. [SRef. 4]

Finally, we discuss the negative susceptibility of LSMO particles[S4]. This experiment provides the susceptibility of nanocomposites with different weight percentages. As we know that the density of LSMO is roughly 6.5 g/cm^3 and the density of epoxy is roughly 1.2 g/cm^3, we can convert the weight percentages into the volume fractions. This can help us to check if our assumption $C = C(v_f) = C_0 v_f$ for some constant C_0 is reasonable. The fitting results are shown in Fig. S3(a)(b)(c). The assumption may be less accurate for the real part but performs well for the imaginary part as shown in Fig. S3(c). Also, Fig. S3(a) shows that the value $\mathrm{Re}(\chi) f / \mathrm{Im}(\chi)$ is roughly the same for all weight percentages with no obvious percentages dependence. Hence, some features of LSMO/epoxy nanocomposites agree with our hypothesis over magnetite/epoxy nanocomposites.

By these three examples, we demonstrate that the model can be applied on variable nanocomposites, and offers a fitting

procedure, which can justify if this model is suitable for a particular composite when operating linear regression.

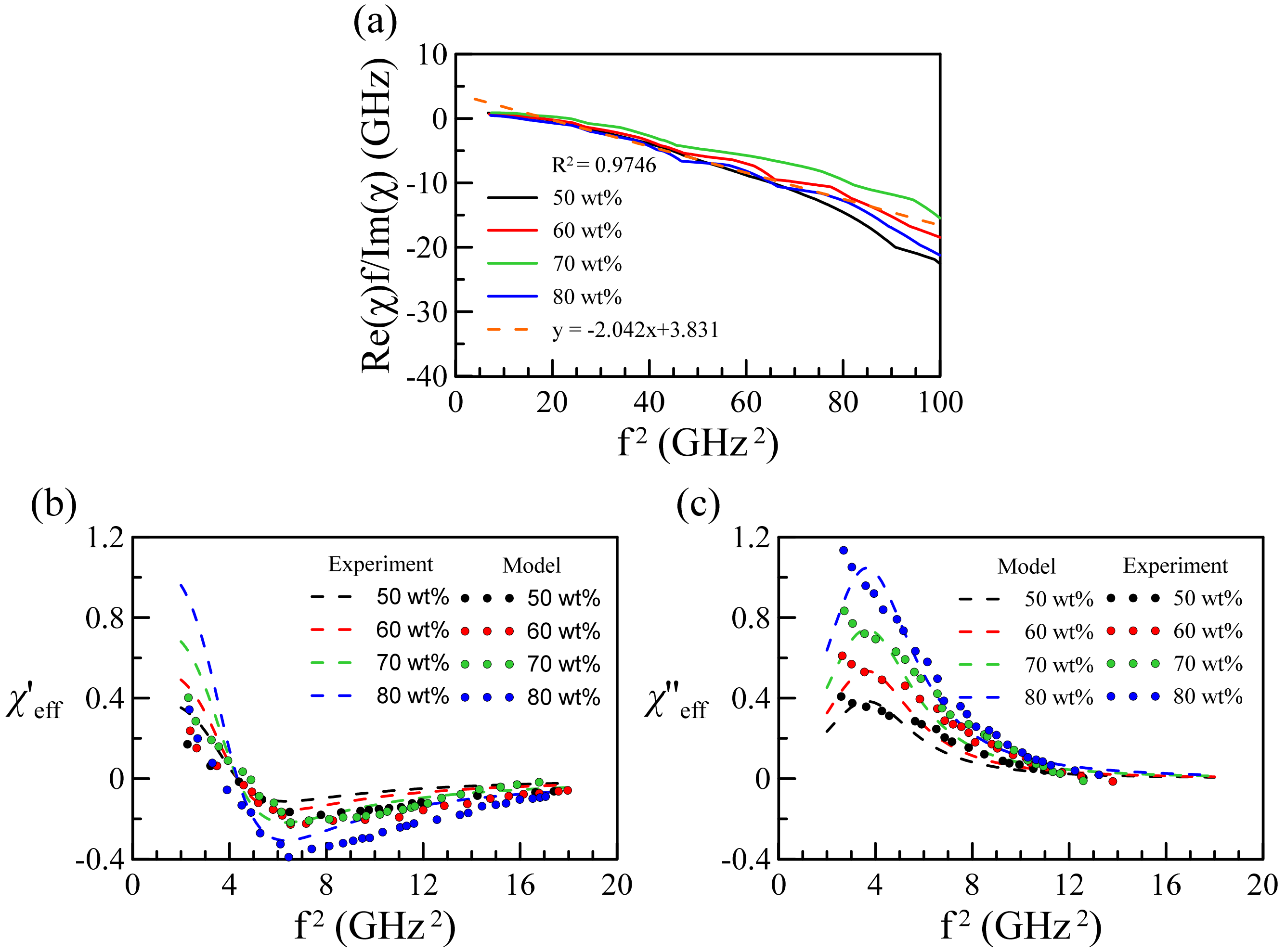

Figure. S3. (a) The linear regression using Eq. (14). (b) the real part and (c) the imaginary part of the magnetic susceptibility of the $FeNi_3$/paraffin nanocomposite from experimental data of SRef. 4 and model fitting, where we use $C = 2.564 v_f$, $\Gamma_f = 3.831\,\text{GHz}$ and $\Theta_f = 0.2042$ ns.

Part V. Further demonstration of transmission/reflection parameters

The transmission/reflection parameters directly from one PNA measurement are shown in Fig. S4. The first number of S-parameters represents where the input signal comes and the second number represents where the output signal is received. Theoretically, the S_{11} parameters and S_{22} should be the same as they are both reflection parameters while S_{21} parameters and S_{12} should be the same as they are both transmission parameters. However, Fig. S4 shows that they are not the same, especially for the reflection parameters of samples with a magnetite volume fraction of 6% and 18%. The differences mainly come from the imperfection of the sample as similar results emerge when repeating measurements.

The complex permittivity and permeability is related to the transmission parameter T and reflection parameter R as[S5]

$$\begin{cases} T = \dfrac{4xe^{ik_2L}}{(x+1)^2-(x-1)e^{i2k_2L}} \\ R = \dfrac{1-x}{1+x}(1-Te^{ik_2L}) \end{cases}, \tag{S10}$$

where k_1 is the wavenumber of TE_{10} mode in the air, k_2 is the wavenumber of TE_{10} mode in the sample, L is the thickness of the sample, and $x = k_1/(k_2\mu_{\text{eff}})$. The thicknesses of samples are 3.996mm, 3.976mm, 3.946mm, and 2.921mm respectively for samples with a volume fraction 0%, 6%, 12%, and 18%. Using this relation, ε_{eff} and μ_{eff} can be analytically deduced from reflection and transmission parameters. In the algorithm, we used S_{11} and S_{21} as a group and use S_{22} and S_{12} as another group to calculate ε_{eff} and μ_{eff} separately. We repeated the measurements and calculation six times and then took the averages and standard deviations from the twelve values of ε_{eff} and μ_{eff}.

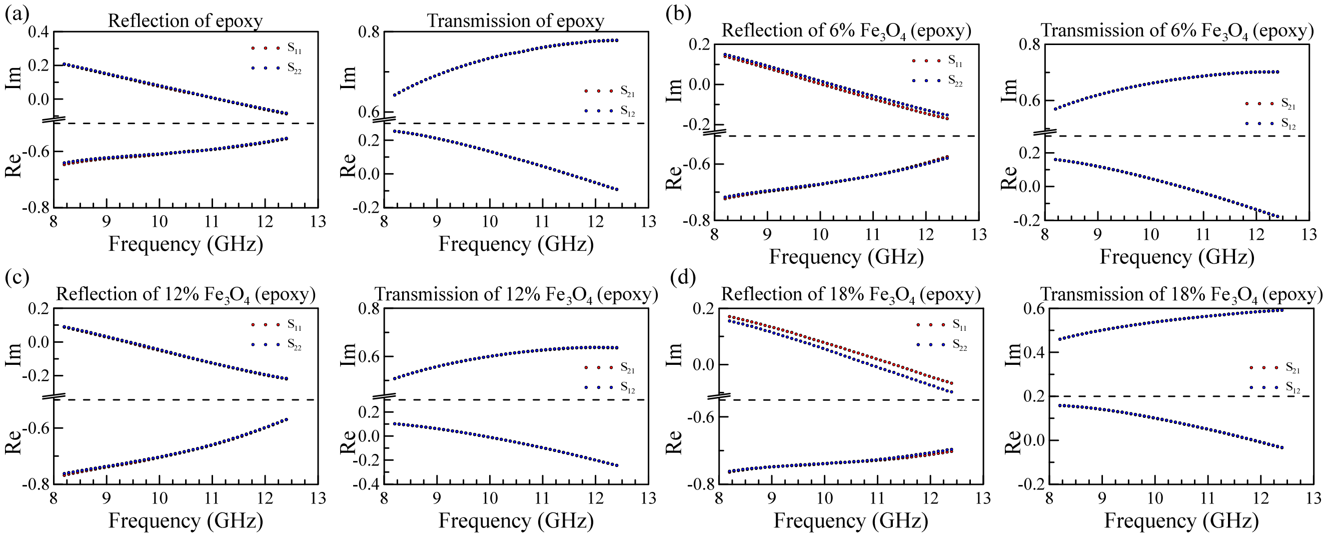

Figure. S4. The transmission/reflection parameters of the sample of (a) pure epoxy (b) magnetite/epoxy with v_f=0.06 (c) magnetite/epoxy with v_f=0.12 (d) magnetite/epoxy with v_f=0.18.

SReference

S1. Miyazaki, K. and Seki, K. Brownian motion of spins revisited. J. Chem. Phys. **108**, 7052-7059 (1998).

S2. Liu, X. G., Ou, Z. Q., Geng, D. Y., Han, Z., Xie, Z. G., & Zhang, Z. D. Enhanced natural resonance and attenuation properties in superparamagnetic graphite-coated $FeNi_3$ nanocapsules. *J. Phys. D: Appl. Phys.* **42**, 155004 (2009).

S3. Liu, J. R., Itoh, M., Horikawa, T., Machida, K. I., Sugimoto, S., & Maeda, T. Gigahertz range electromagnetic wave absorbers made of amorphous-carbon-based magnetic nanocomposites. *J. Appl. Phys.*, **98**, 054305 (2005)

S4. Yang, R. B., Tsay, C. Y., Liang, W. F., and Lin, C. K. Microwave absorbing properties of $La_{0.7}Sr_{0.3}MnO_3$ composites with negative magnetic susceptibility. *J. Appl. Phys.* **107**, 09A523 (2010).

S5. Janezic, M. D., Paulter, N. G., & Blendell, J. E. Dielectric and conductor-loss characterization and measurements on electronic packaging materials. *NIST Technical note*, *1520* (2001).
